# Incidence of emergency contacts (red responses) to Norwegian emergency primary healthcare services in 2007 – a prospective observational study

**DOI:** 10.1186/1757-7241-17-30

**Published:** 2009-07-08

**Authors:** Erik Zakariassen, Elisabeth Holm Hansen, Steinar Hunskaar

**Affiliations:** 1 National Centre for Emergency Primary Health Care, Kalfarveien 31, NO-5018 Bergen, Norway; 2 Department of Research, Norwegian Air Ambulance Foundation, Box 94, NO-1441 Drøbak, Norway; 3 Section for General Practice, Department of Public Health and Primary Health Care, University of Bergen, Kalfarveien 31, NO-5018 Bergen, Norway

## Abstract

**Background:**

The municipalities are responsible for the emergency primary health care services in Norway. These services include casualty clinics, primary doctors on-call and local emergency medical communication centres (LEMC). The National centre for emergency primary health care has initiated an enterprise called "The Watchtowers", comprising emergency primary health care districts, to provide routine information (patients' way of contact, level of urgency and first action taken by the out-of-hours services) over several years based on a minimal dataset. This will enable monitoring, evaluation and comparison of the respective activities in the emergency primary health care services. The aim of this study was to assess incidence of emergency contacts (potential life-threatening situations, red responses) to the emergency primary health care service.

**Methods:**

A representative sample of Norwegian emergency primary health care districts, "The Watchtowers" recorded all contacts and first action taken during the year of 2007. All the variables were continuously registered in a data program by the attending nurses and sent by email to the National Centre for Emergency Primary Health Care at a monthly basis.

**Results:**

During 2007 the Watchtowers registered 85 288 contacts, of which 1 946 (2.3%) were defined as emergency contacts (red responses), corresponding to a rate of 9 per 1 000 inhabitants per year. 65% of the instances were initiated by patient, next of kin or health personnel by calling local emergency medical communication centres or meeting directly at the casualty clinics. In 48% of the red responses, the first action taken was a call-out of doctor and ambulance. On a national basis we can estimate approximately 42 500 red responses per year in the EPH in Norway.

**Conclusion:**

The emergency primary health care services constitute an important part of the emergency system in Norway. Patients call the LEMC or meet directly at casualty clinics with medical problems that initially are classified as a potentially life-threatening situation, a red response.

## Background

In Norway the local municipalities are responsible for the emergency primary health care system. The emergency primary health care system consist of local emergency medical communication centres (LEMC), open 24 hours a day, rGPs, some casualty clinics in office hours and the out-of-hours services. The out-of-hours services consist of casualty clinics and primary care doctors on-call. The emergency primary health care services are served by the LEMCs [1,2]. Intermunicipal cooperatives are common, and in 2006, 433 municipalities were organised into 260 out-of-hours districts with 99 intermunicipal cooperatives and 161 single-municipal out-of-hours districts [3]. The central government is responsible for the secondary health care system, including hospitals, national emergency medical communication centres (EMCC) and the ambulance services. As a rule, in potentially life-threatening problem, a red response, inhabitants shall call the three digit emergency number 113 to an EMCC. If it's less serious, inhabitants shall call the LEMC.

During normal office hours the patients can call their regular general practitioner (rGP) and get an immediate appointment. At all time they can ask for assistance from LEMC and after initial triage be directed to a rGP, a primary care doctor or a casualty clinic. Certain places patients can meet directly at a casualty clinic without an appointment, also on daytime. Furthermore, they can call EMCC and ask for an ambulance. The LEMC can transfer the call to the EMCC when there is a need for an ambulance, or the EMCC can contact the LEMC or casualty clinic if that seems to be the best solution for the patient. The ambulances may transport patients to casualty clinics or directly to hospitals by ground, sea or air transport.

There is little data on regular general practitioners' experience with emergency patients in Norway [4]. The out-of-hours services are organised very differently in different European countries, and there is a lack of reliable data from the services [5,6], in Norway as well. The National Centre for Emergency Primary Health Care has initiated an enterprise called "The Watchtowers" [7]. The purpose of the Watchtower project is to provide routine information (patients' way of contact, level of urgency and first action taken by the out-of-hours services) over several years, based on a minimal dataset, which will enable monitoring, evaluation and comparison of the respective activities in the out-of-hours services. The LEMCs receive calls concerning all grades of medical problems, and triage is carried out based on the Norwegian Index of Medical Emergency Assistance [8]. The most urgent incidences can principally be handled through LEMCs and the emergency primary health care services although transfer of responsibility to EMCCs is common. The emergency primary health care service is the target for the data collection. The aim of this study was to investigate the incidence of red responses in the emergency primary health care services during the first full year of the Watchtower enterprise (2007).

## Methods

The Watchtowers, a representative sample of Norwegian municipalities and emergency primary health care services prospectively recorded all contacts and first responses during 2007. The Watchtowers comprise seven emergency primary health care districts presented as WT1-WT7. Two of the districts are intermunicipal cooperatives; WT6 consists of three municipalities and WT2 consists of ten municipalities. WT7 is a typical town district with casualty clinic open on daytime and inhabitants can attend the clinic without an appointment. WT3 and WT4 are rural areas and the rest are a mixture of rural and more populated areas. The Watchtowers were chosen based on data from Statistics Norway to ensure a representative sample reflecting the emergency primary health care districts in Norway [7]. The Watchtowers had a population of 216,030, which is approximately 5% of the total Norwegian population of 4,681,134 in January 2007.

The following data were collected:

1. Time of contact; week of the year (x/52), day of the week (x/7) and time of the day (daytime 08.00–15.29, afternoon 15.30–22.59 and night 23.00–07.59).

2. Nationality and place of residence (municipality name and number) of the patient.

3. Gender and age of patients. A child of less than one year is registered with the value zero.

4. Mode of contact; telephone contact by patient or next of kin, direct attendance by patient to a casualty clinic, contact by other health personnel, contact through EMCC or others (e.g. police).

5. First action taken, with seven categories; telephone advice by nurse, telephone advice by doctor, medical examination by a primary care doctor on call, consultation by nurse, call-out of a primary care doctor and ambulance, home visit by a primary care doctor and other (e.g. sending ambulance without doctor, referring to police or regular GP on daytime).

6. Priority degree (three levels) according to the Norwegian Index of Medical Emergency Assistance.

All the variables were registered in a data program by the attending nurses and enclosed with a monthly email to the National Centre for Emergency Primary Health Care [7].

Degree of urgency (priority grade) was set according to the Norwegian Index of Medical Emergency Assistance [8]. Each call to or contact with a Watchtower was classified by colour codes "Red", Yellow" or "Green". Red colour was defined as an "acute" response, potentially life-threatening, with the highest priority. Yellow colour was defined as an "urgent" response, with a high, but lower priority. Green colour was defined as a "not urgent" response, with the lowest priority. Age was further categorised 0–9, 10–19, 20–39, 40–59 and ≥ 60 years.

Contacts during daytime to casualty clinics, telephone calls to LEMCs and alarms to rGPs during daytime through the LEMCs together with activity in the out-of-hours services are included in the study. When patients contacted the emergency primary health care services concerning an actual health problem the emergency primary health care services were defined as the primary contact point, in contrast to cases where patients called the three digit emergency number to an EMCC. Contacts through EMCC are exclusively counted when LEMCs are involved in case.

Two casualty clinics lost some cases due to technical problems. The missing data represent one percent of the total and its impact is insignificant on the presented data. Statistical analyses and presentations are therefore solely based on registered data.

### Statistical analyses

All statistical analyses are solely based on red responses and were performed using SPSS version 15. Standard descriptive statistics were used to characterise the data. Rates are presented per 1 000 inhabitants. Normal distributed data are presented as mean (SD). The data constitute a full representation of the population in the Watchtowers and p-values and confidence intervals are not considered to be necessary when the total material is discussed. Differences between variables were analyzed by Pearson's χ^2 ^test. Fisher's exact test was computed when tables had cells with frequency of less than five in 2 × 2 tables. P value < 0.05 was considered as statistical significant. Logistic regression analyses were used to calculate the odds ratio (OR) for different contact forms and odds ratios for relevant alternatives for first responses (consultation by doctor, call-out doctor and ambulance and other responses). The dependent variables were dichotomised (e.g. "mode of contact" into "telephone from patients" vs. "other contact forms" and "first action taken" into "consultation doctor" vs. "other first actions"). Explanatory variables used were gender, age and time of day the contact were made.

## Results

During 2007 the Watchtowers registered 85 288 contacts. Of those, 76.6% were categorised as green, 21.1% as yellow and 2.3% as red responses. Further results and analyses are based on the red responses (N = 1 946). Mean age of patients was 53 (26), range 0–99 years, and 53% were men. Distributions of red responses by age and out-of-hours districts are shown in table 1. The total rate of red responses per 1,000 inhabitants was 9, but varied between districts from 6 to 17. Inhabitants 60 years or older had three to five times higher rates of red responses compared to the other age categories. Rates of red responses were highest during the evenings.

**Table 1:** Red responses (n = 1 946) distributed by districts, age, municipal cooperative, and time of day.

			**Rates**			
			
	**Total**		**Time of day**			
	
**Variable**	n	%	Total	Daytime	Evening	Night
**All (216 030 inhabitants)**	1 946	100	9	3	4	2
**Out-of-hours districts (inhabitants)**						
WT1 (18 090)	303	15	17	7	7	3
WT2 (85 977)	538	28	6	2	3	1
WT3 (4 389)	73	4	17	6	6	5
WT4 (8 230)	72	4	9	3	4	2
WT5 (18 219)	205	10	11	2	6	3
WT6 (16 633)	207	11	12	4	5	3
WT7 (64 492)	548	28	8	3	3	2
Age in year* (inhabitants)						
0–9 (27 553)	118	6	4	2	2	~ 0
10–19 (29 949)	150	8	5	2	2	1
20–39 (57 651)	356	18	6	1	3	2
40–59 (59 490)	438	23	7	2	3	2
60+ (41 357)	856	44	20	7	9	4
**Type of out-of-hours district (inhabitants)**						
Intermunicipal (102 610)	745	38	7	2	4	1
Municipal (113 420)	1 201	62	11	3	5	3

Rate is red responses per 1 000 inhabitants per year.

* Due to missing data age have n = 1 930

Main contact form and first action taken in the different emergency primary health care districts are listed in table S1; Additional file 1. Telephone directly to the emergency primary health care services or direct attendance to casualty clinics counted for 54% of the contacts. Call-out for primary care doctor on-call and ambulance was first action taken in 48% of the cases. Differences between the emergency primary health care districts were large, especially between the least and the most populated districts.

Distributions of first action taken by gender, age, time of day and mode of contact are listed in table S2; Additional file 2. Mode of contact did to some extent predict first action taken. In cases of direct attendance 90% of the patients got a consultation by a doctor. Calls through EMCCs resulted in call-out for a primary care doctor and ambulance or a call-out for ambulance alone in 73% of the cases. Differences were found for the variables gender, age and time of day, but except for the age group 60+ the differences were minor.

The logistic regression analyses support the findings in the descriptive analyses. Age above 60 years had a strong effect on first action taken. Time of day had effect on contacts through the EMCC (table S3; Additional file 3).

National estimates for red responses in Norway are listed in table 2. More than 42 000 (2.3%) contacts to the emergency primary health care service will be categorised as red responses. Two thirds of the patients had the emergency primary health care service as primary contact point.

**Table 2:** National estimates for incidence of red responses in the Norwegian out-of-hours services in 2007 Norwegian population 01.01.2007; 4 681 134

**Variables**	**Numbers**	**%**	**Per 1000**
**Mode of contact in red responses**			
Telephone	17 035	39	4
Direct attendance	6 098	14	1
Health personnel	4 687	12	1
Through EMCC	13 975	33	3
Others	694	2	~ 0
Total	42 489	100	9
**First action taken**			
Consultation by doctor	14 561	35	3
Call out of doctor and ambulance	19 964	48	4
Home visit by doctor	673	2	~ 0
Other	6 749	15	2
Total*	41 947	100	9

*Differences in total numbers between contact and first action taken are due to missing data

## Discussion

Red responses represent less than three percent of the total number of patients who were in contact with the emergency primary health care services in Norway in 2007. Telephone to the emergency primary health care service or LEMC from patients or next of kin and direct attendance were the main contact forms. Only one third of the red responses came through the EMCC. On half of the red responses first action taken was call-out of primary care doctor on-call and ambulance. Patients older than 60 years had the highest rate of red responses.

Data from the Watchtowers are intended to be representative for the whole population and all emergency primary health care districts in Norway [7]. Differences between the emergency primary health care districts in the Watchtower project express variations between emergency primary health care districts in Norway in general.

The fact that more than three out of four patients had minor problems (category green) indicates that many or even a majority of these patients could probably have visited their rGP at daytime, not the emergency primary health care service. In the Netherlands the level of urgent problems was 4.6% for the GP cooperatives [9]. Definition of "urgency" is wider than the definition of "red response" in Norway. However, both the Dutch GP cooperatives and the Norwegian emergency primary health care services are mostly occupied with minor problems. This indicates that there should be a discussion towards more focus on higher priority grades, e.g. more focus on acute and urgent problems.

Evenings have the highest rate of red responses, but regression analyses showed no significant difference for the periods during the day, except for lower probability of calls through the EMCC in the evenings and nights. Emergencies occur 24 hours a day and preparedness cannot be reduced at any time.

In the Netherlands inhabitants can meet directly at hospitals in contrast to Norway where inhabitants first have to attend the primary health care system.

A study from the Netherlands showed more contacts to the ambulance services and direct attendance to accident and emergency departments in the evenings [10]. Another Dutch study showed that when patients called medical attention via accident and emergency departments there were no differences between out-of-hours and office hours [11]. Our regression analysis showed decreasing odds ratios for contacts through the EMCC during evenings and nights A good cooperation between the primary and the secondary health care system is essential to provide patients with good treatment at the appropriate care level.

Main contact form is telephone from patient, next of kin or contact from the EMCC. But there are interesting differences across the Watchtowers. WT7 (typical town district) have a higher proportion of direct attendance, due to casualty clinic with open access. Other districts representing more rural areas or a mix between rural areas and smaller towns have a higher proportion of telephone calls from patients and next of kin. It seems that inhabitants in rural areas tend to call the LEMC or the casualty clinic and inhabitants in city areas tend to call EMCC or meet directly at the casualty clinic. These findings are supported by earlier research [9,12,13]. In small single-municipal emergency primary health care districts, first action taken in the case of almost all red responses was a call-out for doctor and ambulance. Doctors in such districts have been characterised as more ready to act in cases of emergencies compared to doctors in emergency primary health care districts with a higher population [12,14].

The total number of red responses in the ambulance services in 2004 was approximately 119 000 [15]. National estimates based on our research indicate 28 138 red response patients where the emergency primary health care services were the primary contact point (table 2). This strongly indicates that the secondary health care system with their EMCCs does not by far handle all red responses outside hospitals and that the emergency primary health care service make up an important part of the emergency health care system in Norway.

Differences in rates of red responses between the districts could have several explanations. As the oldest inhabitants have higher morbidity and age 60+ had the highest rate of red responses, different age distribution between the out-of-hours districts could be one possible explanation. However, there were no differences in age distribution between the districts. Different structural organisations of the emergency primary health care services can not effect the rate of red responses. But differences in access to rGPs on daytime can influence our data on rates of red responses. We have no data on GPs' accessibility in acute cases during office hours.

Different local triage pattern or traditions of patients are other plausible explanations. The Watchtowers are served by six different EMCCs and nine different LEMCs, and this may explain the differences, even using the same Norwegian Index system. Staff at the casualty clinics will probably not classify patients similarly based on direct attendance compared to telephone triage. Differences in triage, both by telephone and after direct attendance, will also probably exist between the different emergency primary health care districts. Studies on telephone triage demonstrate differences between staff even when using the same guidelines [16], and, not surprisingly, more when using different guidelines [17].

Differences in the number of red responses between the emergency primary health care districts are large. Based on the rate of 9 per 1 000 inhabitants, the largest (Oslo) out-of-hours district in Norway will approximately have 5 000 and the smallest approximately three red responses per year. Better web information about telephone numbers to the LEMCs could increase contact. Telephone numbers to the LEMCs were in half of the municipalities not easily accessible on the Internet [18]. Establishing a common number to the LEMCs in Norway is being discussed. A common phone number will probably increase contacts to the local out-of-hours services [19], underlining the continues need for professional personnel and use of a triage tool with good quality to sort the patients into the right levels of care, also within the local LEMCs and not only the more centralised EMCCs.

## Conclusion

In the emergency primary health care services in Norway, red responses count for less than three percent of all contacts. Still, on a national basis this adds up to more than 42 000 patients per year, out of which only one third is routed through the EMCC. Most patients call the LEMCs or meet directly at casualty clinics. Half of the red responses result in a call-out for a primary care doctor and ambulance. The results emphasise that GP based emergency primary health care service in Norway constitute an important part of the medical emergency system, every hour and day during the year.

## Competing interests

The authors declare that they have no competing interests.

## Authors' contributions

SH planned the project; EHH and SH established the project, including the procedures for data collection. EZ performed the analyses. EZ drafted the manuscript. All authors took part in rewriting and approved the final manuscript.

## Supplementary Material

Additional file 1 **Table S1**. Mode of contact and first action taken in red responses in the Watchtowers out-of-hours districts and distribution (%) of red responses in each out-of-hours district. Click here for file

Additional file 2 **Table S2**. Distributions of first action taken in red responses by gender, age, time of day and mode of contact Click here for file

Additional file 3 **Table S3**. The effect of gender, age and time of day on contact form and first action taken, presented as odds ratios Click here for file
