# Supplementary material for: Incidence of emergency contacts (red responses) to Norwegian emergency primary healthcare services in 2007 – a prospective observational study
Source: Scand J Trauma Resusc Emerg Med. 2009 Jul 8;17:30. doi: 10.1186/1757-7241-17-30 (PMC2725029; doi:10.1186/1757-7241-17-30)
Supplement: Additional file 1 — Table S1. Mode of contact and first action taken in red responses in the Watchtowers out-of-hours districts and distribution (%) of red responses in each out-of-hours district. [file 1757-7241-17-30-S1.doc]

Table 2. Mode of contact and first action taken in red responses in the Watchtowers out-of-hours districts and distribution (%) of red responses in each out-of-hours district.

|  | **WT1** | | **WT2** | | **WT3** | | **WT4** | | **WT5** | | **WT6** | | | **WT7** | | | **Total** | | **p- value** |
| --- | --- | --- | --- | --- | --- | --- | --- | --- | --- | --- | --- | --- | --- | --- | --- | --- | --- | --- | --- |
| **Mode of contact** | **n** | **%** | **n** | **%** | **n** | **%** | **n** | **%** | **n** | **%** | | **n** | **%** | | **n** | **%** | **n** | **%** |  |
| Telephone | 120 | 40 | 244 | 47 | 23 | 32 | 37 | 51 | 71 | 13 | | 136 | 66 | | 127 | 62 | 758 | 39 | < 0.001 |
| Direct attendance | 18 | 6 | 43 | 8 | 1 | 1 | 0 | 0 | 195 | 35 | | 21 | 10 | | 3 | 1 | 281 | 15 | < 0.001 |
| Health personnel | 28 | 9 | 70 | 13 | 11 | 15 | 11 | 15 | 25 | 5 | | 29 | 14 | | 42 | 20 | 216 | 11 | < 0.001 |
| Through EMCC | 130 | 43 | 164 | 31 | 37 | 51 | 17 | 24 | 246 | 45 | | 17 | 8 | | 33 | 16 | 644 | 33 | < 0.001 |
| Others | 6 | 2 | 4 | 1 | 1 | 1 | 7 | 10 | 11 | 2 | | 2 | 2 | | 1 | 1 | 32 | 2 | < 0.001 |
| Total | 302 | 100 | 525 | 100 | 73 | 100 | 72 | 100 | 548 | 100 | | 205 | 100 | | 206 | 100 | 1 931 | 100 |  |
| **First action taken** |  |  |  |  |  |  |  |  |  |  | |  |  | |  |  |  |  |  |
| Telephone consultation by doctor | 6 | 2 | 11 | 2 | 0 | 0 | 2 | 3 | 7 | 1 | | 5 | 2 | | 2 | 1 | 33 | 2 | 0.647 |
| Consultation by doctor | 174 | 58 | 111 | 21 | 0 | 0 | 2 | 3 | 308 | 57 | | 54 | 26 | | 22 | 11 | 671 | 36 | < 0.001 |
| Call out of doctor and ambulance | 104 | 34 | 370 | 69 | 71 | 97 | 66 | 92 | 180 | 34 | | 30 | 15 | | 99 | 48 | 920 | 48 | < 0.001 |
| Home visit by doctor | 0 | 0 | 10 | 2 | 2 | 3 | 1 | 1 | 1 | ~0 | | 8 | 4 | | 10 | 5 | 32 | 2 | < 0.001 |
| Other responds | 17 | 6 | 34 | 6 | 0 | 0 | 1 | 1 | 43 | 8 | | 108 | 53 | | 74 | 35 | 277 | 9 | < 0.001 |
| Total | 301 | 100 | 536 | 100 | 73 | 100 | 72 | 100 | 539 | 100 | | 205 | 100 | | 207 | 100 | 1 933 | 100 |  |

Differences in total numbers between contact and first action taken are due to missing data
