# Supplementary material for: Incidence of emergency contacts (red responses) to Norwegian emergency primary healthcare services in 2007 – a prospective observational study
Source: Scand J Trauma Resusc Emerg Med. 2009 Jul 8;17:30. doi: 10.1186/1757-7241-17-30 (PMC2725029; doi:10.1186/1757-7241-17-30)
Supplement: Additional file 2 — Table S2. Distributions of first action taken in red responses by gender, age, time of day and mode of contact [file 1757-7241-17-30-S2.doc]

Table S2; Additional File 2. Distributions of first action taken in red responses by gender, age, time of day and mode of contact

| **First action taken** | | | | | | | | | | | | | | | | | |
| --- | --- | --- | --- | --- | --- | --- | --- | --- | --- | --- | --- | --- | --- | --- | --- | --- | --- |
|  | Telephone  consultation by doctor | | | Consultation by doctor | | | Call out for doctor and ambulance | | | Home visit by doctor | | | Others | | | Total* | |
|  | n | % | p-value | n | % | p-value | n | % | p-value | n | % | p-  value | n | % | p-value | n | % |
| **Gender** |  |  | 0.87 |  |  | 0.01 |  |  | <0.001 |  |  | 0.20 |  |  | 0.18 |  |  |
| Female | 15 | 2 |  | 286 | 32 |  | 460 | 51 |  | 11 | 1 |  | 125 | 14 |  | 897 | 100 |
| Male | 18 | 2 |  | 385 | 38 |  | 439 | 43 |  | 20 | 2 |  | 153 | 15 |  | 1015 | 100 |
| **Age (years)** |  |  | 0.52 |  |  | <0.001 |  |  | <0.001 |  |  | 0.18 |  |  | 0.10 |  |  |
| 0-9 | 3 | 3 |  | 53 | 46 |  | 44 | 38 |  | 0 | 0 |  | 15 | 13 |  | 115 | 100 |
| 10-19 | 4 | 3 |  | 64 | 43 |  | 64 | 43 |  | 2 | 1 |  | 14 | 10 |  | 148 | 100 |
| 20-39 | 4 | 1 |  | 153 | 43 |  | 141 | 40 |  | 3 | 1 |  | 53 | 15 |  | 354 | 100 |
| 40-59 | 5 | 1 |  | 178 | 41 |  | 184 | 43 |  | 6 | 1 |  | 61 | 14 |  | 434 | 100 |
| 60+ | 17 | 2 |  | 222 | 26 |  | 465 | 54 |  | 20 | 2 |  | 132 | 16 |  | 856 | 100 |
| **Time of day** |  |  | 0.77 |  |  | 0.02 |  |  | 0.47 |  |  | 0.93 |  |  | 0.20 |  |  |
| 08.00-15.29 | 9 | 1 |  | 214 | 34 |  | 309 | 50 |  | 11 | 2 |  | 82 | 13 |  | 625 | 100 |
| 15.30-22.59 | 15 | 2 |  | 325 | 37 |  | 399 | 46 |  | 13 | 2 |  | 114 | 13 |  | 866 | 100 |
| 22.30-07.59 | 9 | 2 |  | 132 | 29 |  | 212 | 48 |  | 7 | 1 |  | 88 | 20 |  | 442 | 100 |
| **Mode of contact** |  |  | 0.28 |  |  | <0.001 |  |  | <0.001 |  |  | <0.001 |  |  | <0.001 |  |  |
| Telephone | 16 | 2 |  | 205 | 27 |  | 347 | 46 |  | 15 | 2 |  | 174 | 23 |  | 757 | 100 |
| Direct attendance | 2 | 1 |  | 247 | 90 |  | 13 | 5 |  | 0 | 0 |  | 12 | 4 |  | 274 | 100 |
| Health personnel | 6 | 3 |  | 39 | 18 |  | 118 | 54 |  | 8 | 4 |  | 45 | 21 |  | 216 | 100 |
| EMCC | 8 | 1 |  | 165 | 25 |  | 421 | 66 |  | 6 | 1 |  | 43 | 7 |  | 642 | 100 |
| Other | 1 | 3 |  | 11 | 35 |  | 13 | 41 |  | 2 | 6 |  | 5 | 15 |  | 32 | 100 |
| *Due to missing data figures under total differs from n= 1 946 | | | | | | | | | | | | | | | | | |
