# Supplementary material for: Incidence of emergency contacts (red responses) to Norwegian emergency primary healthcare services in 2007 – a prospective observational study
Source: Scand J Trauma Resusc Emerg Med. 2009 Jul 8;17:30. doi: 10.1186/1757-7241-17-30 (PMC2725029; doi:10.1186/1757-7241-17-30)
Supplement: Additional file 3 — Table S3. The effect of gender, age and time of day on contact form and first action taken, presented as odds ratios [file 1757-7241-17-30-S3.doc]

Table S3; Additional File 3. The effect of gender, age and time of day on contact form and first action taken, presented as odds ratios

|  |  |  | **Mode of contact** | | | | **First action taken by the out-of-hours services** | | |
| --- | --- | --- | --- | --- | --- | --- | --- | --- | --- |
|  |  |  | Telephone from patients / next of kind | Direct attendance | Contact by health personnel | Through EMCC | Consultation doctor | Call out doctor & ambulance | Other |
|  | **N** | **%** | **OR (95%)** | **OR (95%)** | **OR (95%)** | **OR (95%)** | **OR (95%)** | **OR (95%)** | **OR (95%)** |
| Gender |  |  |  |  |  |  |  |  |  |
| Female | 893 | 47 | 1 | 1 | 1 | 1 | 1 | 1 | 1 |
| Male | 1017 | 53 | 0.84 (0.70-1.02) | 1.66 (1.27-2.16) | 0.79 (0.59-1.06) | 1.07 (0.88-1.30) | 0.80 (0.66-0.97) | 0.75 (0.62-0.90) | 1.24 (0.94-1.64) |
| Age (years) |  |  |  |  |  |  |  |  |  |
| 0-9 | 117 | 6 | 1 | 1 | 1 | 1 | 1 | 1 | 1 |
| 10-19 | 149 | 8 | 0.57 (0.34-0.95) | 0.90 (0.49-1.66) | 1.89 (0.36-9.95) | 1.52 (0.92-2.52) | 1.02 (0.62-1.67) | 1.19 (0.72-1.97) | 0.86 (0.35-2.11) |
| 20-39 | 356 | 18 | 0.69 (0.45-1.06) | 1.19 (0.71-1.98) | 2.63 (0.59-11.6) | 1.03 (0.66-1.61) | 1.02 (0.67-1.56) | 1.10 (0.71-1.70) | 1.31 (0.63-2.72) |
| 40-59 | 434 | 23 | 1.07 (0.71-1.62) | 0.74 (0.44-1.24) | 3.91 (0.92-16.7) | 0.88 (0.57-1.37) | 1.14 (0.75-1.72) | 1.20 (0.79-1.83) | 1.46 (0.72-2.92) |
| ≥60 | 854 | 45 | 0.87 (0.58-1.28) | 0.37 (0.22-0.62) | 13.3 (3.26-54.6) | 0.90 (0.60-1.37) | 2.24 (1.51-3.33) | 1.97 (1.32-2.94) | 1.75 (0.89-3.45) |
| Time of day |  |  |  |  |  |  |  |  |  |
| 08.00-15.29 | 612 | 32 | 1 | 1 | 1 | 1 | 1 | 1 | 1 |
| 15.30-22.59 | 856 | 45 | 1.26 (1.01-1.56) | 1.25 (0.93-1.69) | 1.13 (0.81-1.59) | 0.66 (0.53-0.82) | 0.88 (0.71-1.10) | 0.91 (0.73-1.12) | 0.96 (0.68-1.31) |
| 22.30-07.59 | 442 | 23 | 1.19 (0.93-1.54) | 0.96 (0.67-1.39) | 1.13 (0.75-1.68) | 0.77 (0.59-0.99) | 1.31 (1.00-1.71) | 0.98 (0.77-1.26) | 1.26 (0.88-1.81) |
